# The global use of the International Classification of Diseases to Perinatal Mortality (ICD-PM): A systematic review

**DOI:** 10.7189/jogh.12.04069

**Published:** 2022-08-17

**Authors:** Zita D Prüst, Lachmi R Kodan, Thomas van den Akker, Kitty WM Bloemenkamp, Marcus J Rijken, Kim JC Verschueren

**Affiliations:** 1 Department of Obstetrics, Division Women and Baby, Birth Centre Wilhelmina’s Children Hospital, University Medical Centre Utrecht, Utrecht University, Utrecht, the Netherlands; 2 Department of Obstetrics and Gynaecology, Academic Hospital Paramaribo (AZP), Paramaribo, Suriname; 3 Anton de Kom University of Suriname, Paramaribo, Suriname; 4 Department of Obstetrics and Gynaecology, Leiden University Medical Center, Leiden, the Netherlands; 5 Julius Global Health, The Julius Centre for Health Sciences and Primary Care, University Medical Centre Utrecht, Utrecht University, Utrecht, the Netherlands

## Abstract

**Background:**

The World Health Organization launched the International Classification of Diseases for Perinatal Mortality (ICD-PM) in 2016 to uniformly report on the causes of perinatal deaths. In this systematic review, we aim to describe the global use of the ICD-PM by reporting causes of perinatal mortality and summarizing challenges and suggested amendments.

**Methods:**

We systematically searched MEDLINE, Embase, Global Health, and CINAHL databases using key terms related to perinatal mortality and the classification for causes of death. We included studies that applied the ICD-PM and were published between January 2016 and June 2021. The ICD-PM data were extracted and a qualitative analysis was performed to summarize the challenges of the ICD-PM. We applied the PRISMA guidelines, registered our protocol at PROSPERO [CRD42020203466], and used the Appraisal tool for Cross-Sectional Studies (AXIS) as a framework to evaluate the quality of evidence.

**Results:**

The search retrieved 6599 reports. Of these, we included 15 studies that applied the ICD-PM to 44 900 perinatal deaths. Most causes varied widely; for example, “antepartum hypoxia” was the cause of stillbirths in 0% to 46% (median = 12%, n = 95) in low-income settings, 0% to 62% (median = 6%, n = 1159) in middle-income settings and 0% to 55% (median = 5%, n = 249) in high-income settings. Five studies reported challenges and suggested amendments to the ICD-PM. The most frequently reported challenges included the high proportion of antepartum deaths of unspecified cause (five studies), the inability to determine the cause of death when the timing of death is unknown (three studies), and the challenge of assigning one cause in case of multiple contributing conditions (three studies).

**Conclusions:**

The ICD-PM is increasingly being used across the globe and gives health care providers insight into the causes of perinatal death in different settings. However, there is wide variation in reported causes of perinatal death across comparable settings, which suggests that the ICD-PM is applied inconsistently. We summarized the suggested amendments and made additional recommendations to improve the use of the ICD-PM and help strengthen its consistency.

**Registration:**

PROSPERO [CRD42020203466].

Perinatal death rates and causes reflect a health care system’s strength [1], and perinatal mortality has gained worldwide attention over the last decade, with global actions aiming for its reduction [2,3].

Perinatal death results from a complex pathophysiological interaction between the pregnant woman and her baby, with multiple factors contributing to it. Capturing those numerous factors is challenging when assigning a cause of death by a perinatal death classification system. In response to the many perinatal death classification systems used globally [4-6], the World Health Organization (WHO) developed the International Classification of Disease Perinatal Mortality (ICD-PM) in 2016 [7]. The ICD-PM was designed to uniformly identify causes and harmonize perinatal mortality data globally.

The ICD-PM tool requires the identification of the timing of death, the cause of death, and the associated maternal condition [7]. The tool was validated following a pilot study on databases from South Africa and the United Kingdom [8]. Five years after the introduction of the ICD-PM, the number of studies investigating perinatal deaths using the ICD-PM increased rapidly in low-, middle-, and high-income settings [8-10]. The overall results and applicability of the tool across these settings have not yet been evaluated.

In this systematic review, we aim to evaluate the global use of the ICD-PM tool by reporting causes of perinatal death across low-income, middle- and high-income settings and summarizing the challenges and suggested amendments.

## METHODS

This systematic review was conducted using the Cochrane Collaboration principles and the Preferred Reporting Items for Systematic reviews and Meta-Analysis (PRISMA) guideline [11,12]. The study protocol was registered in PROSPERO [CRD42020203466].

### Search strategy and study selection

We conducted a systematic search to identify all studies that applied the ICD-PM. Studies were included if they classified the causes of stillbirths and/or neonatal deaths according to the ICD-PM between January 1, 2016, and June 1, 2021. Studies were excluded if 1) a classification system other than the ICD-PM was used to identify causes of death; 2) no original data was reported; 3) there was no full text available, or; 4) the ICD-PM classification data could not be extracted from the presented text, tables, or additional files.

We searched the MEDLINE, Embase, Global Health, and CINAHL databases. The authors (ZP, LK, KV) established the key search terms in consultation with a medical librarian with expertise in systematic review searching. We searched using terms related to perinatal mortality and terms related to the classification of the causes of death (see file S1 in the **Online Supplementary Document**). After a systematic search of the databases, we assessed grey literature sources and used Scopus for reference and citation checking.

### Data collection

**Figure 1:**
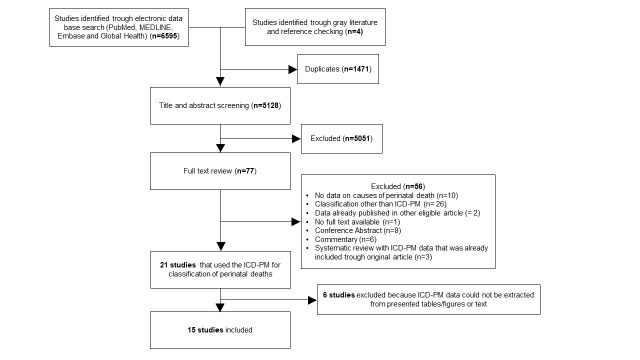
Flowchart of study selection.

We removed duplicates by using the Zotero reference manager database and imported the articles to Rayyan online software for systematic reviews [13,14] Three independent reviewers (ZP, KV, LK) screened the titles, abstracts and full texts . The authors recorded their reasons for excluding ineligible studies. We resolved disagreements by consulting other authors (KB, MR, TA). The selection process was recorded in a PRISMA flow diagram (**Figure 1**), and the characteristics of the excluded studies were summarized in Table S2 of the **Online Supplementary Document****).**

We manually extracted data into Microsoft Excel based on a standard data collection form adapted from the Cochrane good practice data collection form (see file S3 in the **Online Supplementary Document**). We extracted study characteristics (eg, study design and eligibility criteria), timing of death (antepartum, intrapartum, or neonatal), cause of death (one of the 24 ICD-PM categories), correlated maternal conditions (one of the six ICD-PM maternal condition categories) and, if reported, the perinatal mortality rate (PMR), the neonatal mortality rate (NMR) and the stillbirth rate (SBR) of all eligible studies (Table S3 in the **Online Supplementary Document**) [7]. We extracted the reported challenges related to the use of the ICD-PM and the amendments the authors suggested to improve the ICD-PM.

### Quality assessment tool

Three review authors (ZP, KV, and LK) independently assessed the methodological quality using the Appraisal tool for Cross-Sectional Studies (AXIS, 2016) [15]. It includes 20 items, each containing one point (0 or 1). If an item was not applicable to the article, it was not scored (filled in as Not Applicable (N.A.)). The final score was calculated by dividing the obtained score with the total points possible after withdrawing the number of items that were not applicable for the article. This resulted in a final score between 0 and 1, categorized into weak (<0.5), moderate (0.51-0.65), moderate-strong (0.66-0.79) or strong (>0.80) methodological quality [16]. See Table S4 in the **Online Supplementary Document** for the full assessment.

### Data analysis

Descriptive statistics (percentage, medians) were used to present the timing and causes of perinatal death and the associated maternal conditions. We calculated the PMR (perinatal deaths per 1000 total births), consisting of the NMR (neonatal deaths per 1000 live births) and the SBR (stillbirths per 1000 total births) using medians. We used Microsoft Office Excel to synthesize the data. The ICD-PM classification and mortality ratios were synthesized in tables. If the ICD-PM classification data could not be extracted from the presented text, tables, or additional files, the study was excluded from the cause of death analysis. The reported challenges and recommendations to improve ICD-PM applicability were summarized in textboxes.

## RESULTS

Our search yielded 6599 citations from January 2016 to June 2021. **Figure 1** illustrates the study selection through the different phases of this systematic review. In the end, 15 studies were included.

Using the quality assessment tool, eight studies were rated as strong [17-24], three as moderately strong [9,25,26], three as moderate [10,27,28], and one as weak [8] (**Table 1****).** We could not assess the sample size justification and non-responders for most studies in this review (see table S4 in the **Online Supplementary Document**).

**Table 1:** AXIS Quality assessment of the included studies

Author, year	Introduction (1 point)	Methods (10 points)	Results (5 points)	Discussion (2 points)	Other (2 points)	Total points	Score	Quality
Allanson, 2016 [8]	1	2	2	1	1	7/17	0.41	Weak
Lavin, 2018 [25]	1	6	2	1	2	12/17	0.71	Moderate – strong
Aminu, 2019 [9]	1	9	3	1	1	15/19	0.79	Moderate – strong
Madhi, 2019* [27,28]	1	4	2	2	1	10/18	0.56	Moderate
Miyoshi, 2019 [10]	1	4	2	1	2	10/17	0.59	Moderate
Salazar-Barrientos, 2019 [20]	1	7	3	2	1	14/17	0.82	Strong
Dase, 2020 [18]	1	6	3	2	2	14/17	0.82	Strong
Fabrizio, 2020 [21]	1	8	3	2	1	15/17	0.88	Strong
Luk, 2020 [23]	1	6	3	2	2	14/17	0.82	Strong
Prüst, 2020 [19]	1	8	3	2	1	15/17	0.88	Strong
Shattnawi, 2020 [24]	1	6	4	2	1	14/17	0.82	Strong
Wasim, 2020 [17]	1	8	3	1	1	14/17	0.82	Strong
Housseine, 2021 [26]	1	6	2	2	2	13/17	0.76	Moderate – strong
Sharma, 2021 [22]	1	7	3	2	2	15/17	0.88	Strong

*Includes two published studies by authors Madhi et al. reporting one study population [27,28].

The 15 included studies conducted in 16 countries comprised 45 735 perinatal deaths. **Table 2** summarizes the characteristics of the included studies per setting. 61% (n = 7781) of the perinatal deaths occurred in South Africa [8,25,27,28]. The ICD-PM was applied to 44 900 perinatal deaths (**Table 3**). Of these, 29 672 (66%) were stillbirths, 14 609 (33%) neonatal deaths, and 619 (1%) perinatal deaths of unknown timing. Of all perinatal deaths classified by the ICD-PM, 2556 (6%) occurred in low-income countries, 32 715 (73%) in middle-income countries, and 9629 (21%) in high-income countries.

**Table 2:** Characteristics of the included studies

	Author, year	Country	Centre(s)	Design	Classification system(s)	No.	Study population		SBR††	NMR‡‡	PMR††
**Low-income**							Stillbirths	Neonatal deaths			
	Aminu, 2019 [9]	Kenya, Malawi, Zimbabwe, Sierra Leone	12 different hospitals both tertiary and secondary level	Prospective	ICD-PM	K = 321, M = 299, Z = 307, SL = 340, Total = 1267	Yes	No	K = 38.8, M = 20.3, Z = 34.7, SL = 118.1	-	-
	Miyoshi, 2019 [10]	Zambia	One referral hospital	Retrospective	ICD-PM	75	Yes	Yes║	18.2	18.0	35.0
	Dase, 2020 [18]	Nigeria	One tertiary Hospital	Retrospective	ICD-PM	1177	Yes	No	55	-	-
	Housseine, 2021 [26]	Tanzania	One tertiary hospital	Prospective	ICD-PM	661	Yes	Yes**	44	27	71
**Middle-income**	Allanson, 2016 [8]*	South Africa (SA),	Population-based (one province)	Retrospective	ICD-PM	689	Yes	Yes	-	-	-
	Lavin, 2018 [25]	South Africa	Nationwide (all 588 clinics across the country)	Retrospective	ICD-PM and the South African perinatal mortality audit system	26810	Yes	Yes	-	-	-
	Madhi, 2019 [27,28]†	South Africa	One Tertiary Hospital	Prospective	ICD-PM	282	Yes	Yes║	-	-	-
	Salaraz-Barrientos, 2019 [20]	Colombia	Population-based (one province)	Retrospective	ICD-PM	3901	Yes‡	Yes║	-	-	13.0i
	Prüst, 2020 [19]	Suriname	Nationwide (five hospitals)	Retrospective	ICD-PM	131	Yes	No	14.1	-	-
	Shattnawi, 2020 [24]	Jordan	Five hospitals (three secondary public, one private, and one tertiary)	Prospective	ICD-PM	102	Yes§	No	9.9	-	-
	Wasim, 2020 [17]	Pakistan	One tertiary hospital	Prospective	ICD-PM	690	Yes§	Yes║	20.3	38.8	58.2
	Sharma, 2021 [22]	India	One tertiary Hospital	Prospective	ICD-PM and CODAC	314	Yes¶	No	54	-	-
**High-income**	Allanson, 2016 [8]*	United Kingdom	Population-based (West Midlands)	Retrospective	ICD-PM	9067	Yes§	Yes║	-	-	-
	Fabrizio, 2020 [21]	Italy	Population-based (All hospitals in the Emilia–Romagna Region)	Prospective	ICD-PM, CODAC and ReCoDe	450	Yes‡	No	3.2	-	-
	Luk, 2020 [23]	Hong Kong, China	One regional public Hospital	Mixed-method: retrospective and prospective	ICD-PM and a Local (simplified) classification system	119	Yes§	Yes║	2.6	0.8	3.4

K – Kenya, M – Malawi, NMR – neonatal mortality rate, PMR – perinatal mortality rate, SBR – stillbirth rate, SL – Sierra Leone, Z – Zimbabwe

*Data sets from the same study [8].

†Includes two published studies by authors Madhi et al. reporting one study population [27,28].

‡≥500 g and/or 22 weeks of gestation.

§≥24 weeks of gestation.

¶≥500 g and/or 20 weeks of gestation.

║Up to 28 d of life.

**Neonatal deaths who died before discharge from the hospital (minimal BW of 1000 g).

††Per 1000 total births.

‡‡Per 1000 live births.

**Table 3:** Causes of perinatal death according to the ICD-PM*

		High-income settings		Middle-income settings		Low-income settings	
	Number of studies	3		9		4	
	Countries	United Kingdom, Italy, Hong Kong		South Africa, Colombia, Suriname, Jordan, Pakistan, India		Sierra Leone, Zimbabwe, Kenya, Malawi, Nigeria, Zambia, Tanzania	
	Total Inclusions (n)	9629		32 715		2556	
	Antepartum stillbirths	4880		17 897		825	
	Intrapartum stillbirths	488		4390		1192	
	Neonatal deaths	4260		10 058		291	
	Timing unknown	1		370		248	
		**Median**	**Range**	**Median**	**Range**	**Median**	**Range**
**Main causes of antepartum deaths (n)**	A1 Congenital malformations, deformations, and chromosomal abnormalities	21	6-22	4	2-20	3	2-14
	A2 Infection	1	0-8	3	0-44	4	0-9
	A3 Antepartum hypoxia	5	0-55	6	0-62	12	0-46
	A4 Other specified antepartum disorder	3	2-4	17	1-19	0	0
	A5 Disorder related to foetal growth	14	6-19	9	1-20	25	0-57
	A6 Foetal death of unspecified cause	57	22-60	61	2-68	57	30-89
**Main causes of intrapartum deaths (n**	I1 Congenital malformations, deformations, and chromosomal abnormalities	3	3-100	7	3-29	4	2-16
	I2 Birth trauma	1	0-1	0	0	0	0
	I3 Acute intrapartum event	64	0-65	67	0-94	29	10-84
	I4 Infection	1	0-17	1	0-22	2	0-4
	I5 Other specified intrapartum disorder	0	0	11	0-29	0	0-0.3
	I6 Disorders related to foetal growth	5	0-5	4	0-49	13	0-40
	I7 Intrapartum death of unspecified cause	25	0-26	9	0-43	51	0-61
**Main causes of neonatal deaths (n)**	N1 Congenital malformations, deformations, and chromosomal abnormalities	27	15-27	13	5-29	7	2-8
	N2 Disorder related to foetal growth	0	0-4	2	0-4	0	0
	N3 Birth trauma	0	0	0	0	0	0
	N4 Complications of intrapartum events	2	2-4	23	0-29	40	40-44
	N5 Convulsions and disorders related to cerebral status	1	0-1	1	0-26	0	0
	N6 Infection	2	2-15	7	2-27	5	5-7
	N7 Respiratory and cardiovascular disorders	7	7-11	20	3-35	13	5-14
	N8 Other neonatal conditions	3	2-19	6	1-10	1	0-1
	N9 Low birthweight and prematurity	32	33-32	26	10-53	13	9-37
	N10 Miscellaneous	0	0	2	0-2	0	0
	N11 Neonatal death of unspecified cause	27	0-27	0	0-2	20	5-23
**Main maternal condition (n)**	M1 Complications of placenta, cord, and membranes	26	24-71	17	13-34	23	6-27
	M2 Maternal complications of pregnancy	10	2-15	4	2-13	8	6-10
	M3 Other complications of labour and delivery	8	0-8	14	2-18	19	9-44
	M4 Maternal medical and surgical conditions	8	7-22	30	6-50	23	4-42
	M5 No maternal condition identified	48	16-50	35	16-57	28	9-55
	M1 Complications of placenta, cord, and membranes	26	24-71	17	13-34	23	6-27

*All values for the median, mean and range in this table are expressed in percentages.

**Table 3** and Table S5 in the **Online Supplementary Document** report the causes of perinatal deaths per setting. Antepartum stillbirth was most frequently of “unspecified cause” (A6), in low- (57%, n = 468/825), middle- (61%, n = 10 851/17 897), and high-income settings (57%, n = 2765/4880). The causes “antepartum hypoxia” (A3) and “acute intrapartum event” (I3) ranged greatly among and between settings. Birth trauma was reported only five times for intrapartum deaths (I2) and 13 times for neonatal deaths (N3) (0%-1% in all settings). Neonatal causes of death differed between the settings. “Complication of intrapartum events” (N4) was the most common cause of neonatal deaths in low-income countries (40%, n = 117/291). “Low birth weight and prematurity” (N9) was the most common cause of neonatal deaths in middle-income countries (26%, n = 2611/10,564) and high-income countries (32%, n = 1347/4260).

**Table 4** summarizes the challenges in the application of the ICD-PM tool reported in five studies [9,19,22,25,26]. The most frequently reported challenges were the high proportion of antepartum deaths of unspecified cause (five studies), the inability to determine the cause of death when the timing of death is unknown (three studies), and the challenge of assigning one cause in case of multiple contributing conditions (three studies). In **Table 4****,** the amendments that authors suggested for the improvement of the tool’s future applicability are added to the challenges [9,19,22,25,26].

**Table 4:** Suggested amendments to the use of ICD-PM which may improve applicability

Reported challenge	Case example or explanation	Suggested amendments
Difficulty in assigning the timing of death [22,26].	If competing information was reported about the foetal heartrate, the progress of birth and the maceration of a stillborn baby.	Inclusion of a standardised definition of antepartum and intrapartum death in the ICD-PM guideline, and recommendations on how to classify if the timing is unclear.
Inability to determine cause of death and maternal condition for deaths of unknown timing [9,19,26].	If the timing of death remains unknown, it is not possible to assign the cause of death or the maternal condition.	Development of a new category for causes of perinatal deaths of unknown timing (eg, as illustrated by Aminu et al.) [9].
High proportion of antepartum deaths of unspecified cause [9,19,22,25,26].	A high proportion of antepartum deaths of unspecified cause was found in all five articles that described challenges of the ICD-PM. This might be due to missing data and a lack of diagnostic assessment of both mother and foetus	Most perinatal death classification systems report a high proportion of unexplained antepartum deaths (not only the ICD-PM). The addition of a diagnostic work-up checklist (cultures, maternal blood work, placenta histology) may improve the attribution of causes.
High proportion of intrapartum deaths of unknown cause [26].	A high proportion of intrapartum deaths of unspecified cause could be related to suboptimal quality of intrapartum care.	The addition of a separate category for modifiable causes, for example according to the three-delay system (patient, transport, health system).
Difficulty in distinguishing between maternal and foetal conditions [19].	Certain conditions, such as a prolapsed cord or breech delivery, are classified as a maternal (instead of a foetal) condition. This is debatable, and the authors argue that these are often not a maternal condition.	Re-evaluate which conditions/events should be considered a maternal complication.
Multiple contributing conditions, variable interpretation of the cause of death [19,25,26].	Many perinatal deaths follow a chain of events with multiple contributing factors. Therefore, the attributed cause can be anywhere between the first and the last event. This leads to inconsistent classification and globally incomparable data. For example, a growth restricted foetus of a mother with pre-eclampsia dies due to asphyxia following a placental abruption before labour, can be classified as A3 ‘antepartum hypoxia’, A4 ‘Other specified antepartum disorder’, or A5 ‘Disorder related to foetal growth’ [29-31].	Recommendations need to clarify where in the chain of events the cause of perinatal death should be attributed.
Difficulty in assigning “disorder related to foetal growth” among stillbirths [9,19].	The gestational age is often uncertain in settings where women do not receive routine first-trimester ultrasounds.	No specific recommendations made for the ICD-PM, as all classification systems face this challenge.
Two different ICD-PM codes for the same cause of death [25].	For example, unspecified, antepartum stillbirth could be coded as either A3 ‘antepartum asphyxia’ or A6 ‘unspecified cause of death’.	If the cause of perinatal death is unknown, it should be classified as A6 ‘Unspecified cause of death’.
Potential for misclassification [9].	For example, it is difficult to associate antepartum stillbirth with ‘other complications of labour and delivery’ (M3) since an antepartum death will by definition have occurred before labour and therefore have little to do with events during labour or birth.	New guidelines for the application of the ICD-PM should highlight this and other potential pitfalls.

## DISCUSSION

The ICD-PM is increasingly applied to classify causes of perinatal mortality around the globe. Our systematic review shows that the reported causes of perinatal deaths vary widely, also across similar settings, which suggests that the tool is used inconsistently. Five studies have reported challenges related to the use of the ICD-PM, suggesting a total of nine amendments. Frequently reported challenges were the high proportion of antepartum deaths of unspecified cause, the inability to determine the cause of death if the timing is unknown, and the challenge of assigning one cause of death when there are multiple contributing conditions.

Perinatal deaths are declining too slowly worldwide, so global goals targets have been set to reduce these deaths more rapidly [2,3,32]. Five years after the ICD-PM was developed, we identified several studies that applied the tool to perinatal death data. By using the ICD-PM, these studies facilitate essential insight into perinatal death causes and helped raise global awareness of perinatal mortality and the complexity of classifying its causes. The ICD-PM is currently considered the gold standard and is often favoured over other classification tools because it facilitates the lowest rate of unspecified causes and because it is the only tool that classifies death by time of death and separates maternal and foetal conditions into two entities [21-23,25,29,30].

The ICD-PM was developed to harmonize perinatal death data and identify patterns in the causes of deaths across comparable settings. Previous perinatal death reports describe such patterns: it shifts from a high proportion of intrapartum deaths due to hypoxia and infection in low-income countries to a high proportion of antepartum deaths due to congenital anomalies and placental conditions in high-income countries [33,34]. Our review, however, does not identify a similar pattern. The heterogeneity among ICD-PM studies may be caused by multiple factors, most importantly the causes of death and maternal conditions, which were classified inconsistently among studies.

First, studies were heterogenous because the causes of death were classified inconsistently. For example, where one study assigned an A5 “disorder related to foetal growth” for every case of unexplained stillbirth with a birthweight <2500 g [18]; other studies assigned an A5 “disorder related to foetal growth” only to cases with a deflection of the birthweight percentile (>20) or a birth weight under the third percentile [19,21].

Another example is that the A3 category “antepartum hypoxia” was interpreted in different ways and assigned when there was placental abruption, perinatal death after severe pre-eclampsia, or even antepartum death of an unknown cause [8,19,25]. However, other studies classified these diagnoses into other groups, such as A4 “other specified antepartum disorders” for placental abruption and A6 “unspecified” for antepartum death of unknown cause [25]. This led to “antepartum hypoxia” being the cause of perinatal death in up to 50% of all cases in some studies [8,19], whilst other studies in similar settings did not classify any cases as ‘antepartum hypoxia’ [9,25]. Additionally, “hypoxia” could be considered the “mode of death” associated with a multitude of underlying causes. It may even be argued that every perinatal death ultimately results from “hypoxia”. The “hypoxia” category can thus be interpreted in different ways, making it difficult to understand and complicating the identification of interventions to improve perinatal outcomes [7,19].

Second, ICD-PM studies were heterogeneous because maternal conditions were classified inconsistently. Studies particularly classified the maternal conditions inconsistently when maternal conditions were mild and not directly linked to the primary cause of death [19]. An example is well-controlled gestational diabetes, which may be classified as M4 ‘maternal surgical and medical condition’ or M5 ‘no maternal condition’ [35]. Comparable to the maternal death classification system, we suggest distinguishing leading causes from contributing factors in the ICD-PM.

We are convinced that integration of maternal death and near-miss classification tools (ICD – Maternal Mortality and WHO – Maternal Near miss) with the ICD-PM would strengthen global applicability and feasibility and lead to more efficient use of resources [31,36].

Based on our study findings, we suggest nine amendments to the ICD-PM to improve its use and enhance the consistency of results (**Box 1**).

Box 1 **Suggested amendments based on our study results.**

1. Include a standardised definition of antepartum and intrapartum deaths in the ICD-PM Guideline and develop a new category for causes of perinatal deaths of unknown timing [9,19,22,25,26].

2. Re-evaluate the “hypoxia” category and, if used, develop a clear explanation and establish guidelines on what conditions should or should not be classified as “hypoxia”.

3. Elaborate recommendations on how to classify perinatal death causes and wherein the chain of events classification should be done [19,25,26].

4. Provide further guidance on when to classify something as a maternal condition, and how to distinguish between cause and contributing factor [19].

5. Highlight potential pitfalls of the ICD-PM in the new guidelines [9].

6. Add a diagnostic work-up checklist for after a perinatal death took place [19].

7. Consider ‘birth trauma’ as ‘subcategory’ instead of ‘main category’.

8. Create a link between the ICD-PM and the WHO ICD-MM and MNM tools.

9. Develop an additional category for modifiable causes [26].

This study is the first to systematically review the applicability of the ICD-PM. The strengths of this study are the use of a wide search in multiple digital databases, the screening of title, abstract, and full text by at least two independent authors, and the use of a validated quality assessment tool to evaluate methodological quality.

The limitations of this study were the exclusion of six articles from which the ICD-PM data could not be extracted, although all the authors concerned were contacted by email to collect the data. Furthermore, 61% of the included perinatal deaths took place in one country (South Africa), which influences the validity of our findings. Finally, we were unable to perform an agreement analysis of the diagnosis in the different studies because the studies did not provide fully explain how the investigators applied the ICD-PM to different diagnoses.

## CONCLUSION

The ICD-PM is increasingly being used worldwide and gives health care providers insight into the causes of perinatal death in different settings. However, our report suggests that the ICD-PM is applied inconsistently, which diminishes the comparability of results. We suggest nine ways to amend the ICD-PM, some of which are the development of a category for deaths of unknown timing, the re-evaluation the “hypoxia” category, the expansion of guidance on how to classify perinatal death causes and maternal conditions, and the inclusion potential pitfalls of the ICD-PM within the next official guidelines.

## Additional material


Online Supplementary Document
