# Supplementary material for: The global use of the International Classification of Diseases to Perinatal Mortality (ICD-PM): A systematic review
Source: J Glob Health. 2022 Aug 17;12:04069. doi: 10.7189/jogh.12.04069 (PMC9380964; doi:10.7189/jogh.12.04069)
Supplement: Online Supplementary Document [file jogh-12-04069-s001.pdf]

Online supplementary Document

Title: The global use of the International Classification of Diseases to Perinatal Mortality (ICD-PM): a systematic review

Authors: Zita D Prüst, Lachmi R Kodan, Thomas van den Akker, Kitty W M Bloemenkamp, Marcus J Rijken, Kim J C Verschueren

Table of content

1. Supplementary file 1: Search strategy…………………………………………………………...2 2.
Supplementary Table 2: Characteristics of excluded studies from full text screening………..4 3.
Supplementary file 3: Data collection form…………………………………………………………10 4.
Supplementary Table 4: Quality assessment of included articles, AXIS tool …………………..16 5.
Supplementary Table 5: Elaborated table on the causes of perinatal deaths according to the ICD-PM, per income setting……………………………………..…………….17
6. References…………………………………………………………………………………………19

Supplementary file 1. Search strategy

PUBMED

"Perinatal Mortality"[MeSH Terms] OR "Perinatal Death"[MeSH Terms] OR "Stillbirth"[MeSH Terms] OR stillbirth*[Title/Abstract] OR still birth*[Title/Abstract] OR ((perinatal[Title/Abstract] OR peri-natal[Title/Abstract] OR late fetal[Title/Abstract] OR late foetal[Title/Abstract] OR "infant, newborn"[MeSH Terms] OR neonat*[Title/Abstract] OR newborn*[Title/Abstract] OR new born*[Title/Abstract] OR "late pregnanc*"[Title/Abstract]) AND ("Mortality"[MeSH Terms] OR "Mortality"[MeSH Subheading] OR mortalit*[Title/Abstract] OR death*[Title/Abstract] OR dead*[Title/Abstract]))

AND

("Classification"[MeSH Terms] OR "Classification"[MeSH Subheading] OR "International Classification of Diseases"[MeSH Terms] OR classify[Title/Abstract] OR classifying[Title/Abstract] OR classification*[Title/Abstract] OR ICD-PM[Title/Abstract] OR framework*[Title/Abstract])

2016:2021[DP]
Date of search: June 1st, 2021

Results: 1884

EMBASE

(exp perinatal mortality/ OR exp perinatal death/ OR exp stillbirth/ OR stillbirth*.ti,ab,kw. OR still birth*.ti,ab,kw. OR ((perinatal.ti,ab,kw. OR peri-natal.ti,ab,kw. OR late fetal.ti,ab,kw. OR late foetal.ti,ab,kw. OR exp newborn/OR neonat*.ti,ab,kw. OR newborn*.ti,ab,kw. OR new born*.ti,ab,kw. OR late pregnanc*.ti,ab,kw.) AND (exp mortality/ OR mortalit*.ti,ab,kw. OR death*.ti,ab,kw. OR dead*.ti,ab,kw.)))

AND
(exp classification/ OR exp International Classification of Diseases/ OR classification*.ti,ab,kw. OR classify.ti,ab,kw. OR classifying.ti,ab,kw. OR framework*.ti,ab,kw. OR ICD-PM.ti,ab,kw.)

2016:2021.(sa_year)
Date of search: June 1st, 2021

Results: 4339 (including duplicates)

CINAHL

(MH "Infant Mortality" OR MH "Perinatal Death" OR TI stillbirth* OR AB stillbirth* OR TI still birth* OR AB still birth* OR ((TI perinatal OR AB perinatal OR TI peri-natal OR AB peri-natal OR TI late fetal OR AB late fetal OR TI late foetal OR AB late foetal OR MH "Infant, Newborn+" OR TI neonat* OR AB neonat* OR TI newborn* OR AB newborn* OR TI new born* OR AB new born* OR TI "late pregnanc*" OR AB "late pregnanc*") AND ("MH "Mortality+"" OR TI mortalit* OR AB mortalit* OR MH "Death+" OR TI death* OR AB death* OR TI dead* OR AB dead*)))

AND

(MH "Classification+" OR MH "International Classification of Diseases" OR TI classification* OR AB classification* OR TI classify OR AB classify OR TI classifying OR AB classifying OR TI ICD-PM OR AB ICD-PM OR TI framework* OR AB framework*)

Date of search: June 1st, 2021

Results (2016-2021): 675

GLOBAL HEALTH

"perinatal mortality" OR "stillbirth" OR title:(stillbirth) OR ab:(stillbirth) OR title:(still birth) OR ab:(still birth) OR (((title:(perinatal) OR ab:(perinatal) OR title:(peri-natal) OR ab:(peri-natal) OR title:(late fetal) OR ab:(late fetal) OR title:(late foetal) OR ab:(late foetal) OR "newborn infants" OR "neonates" OR title:(neonat*) OR ab:(neonat*) OR title:(newborn*) OR ab:(newborn*) OR title:(new born*) OR ab:(new born*) OR title:(late pregnanc*) OR ab:(late pregnanc*)) AND ("death" OR "mortality" OR title:(mortalit*) OR ab:(mortalit*) OR title:(death*) OR ab:(death*) OR title:(dead) OR ab:(dead)))

AND

"classification" OR title:(International Classification of Diseases) OR ab:(International Classification of Diseases) OR title:(classification*) OR ab:(classification*) OR title:(classify) OR ab:(classify) OR title:(classifying) OR ab:(classifying) OR title:(ICD-PM) OR ab:(ICD-PM) OR title:(framework) OR ab:(framework)

Date of search: June 1st, 2021

Results (2016 – 2021): 580 articles

Supplementary Table S2. Characteristics of excluded studies from full text screening

Author, year	Title	Study design	Study aims	Exclusion based on:
**Alfirevic, 2017**	Reducing the impact of perinatal death – The case for increased understanding of underlying causes to inform change to save babies' lives	Commentary	Improve understanding of the key aspects of care related to stillbirth and neonatal deaths	No reporting of primary data
**Allanson, 2016**	Classifying cases of perinatal death	Editorial	Announcing ICD-PM	No reporting of primary data
**Allanson, 2016**	Giving a voice to millions: developing the WHO application of ICD-10 to deaths during the perinatal period: ICD-PM	Commentary	Announcing ICD-PM	No reporting of primary data
**Allanson, 2016**	Optimizing the International Classification of Diseases to identify the maternal condition in the case of perinatal death	Population based, retrospective application to two perinatal death databases (UK and South Africa)	Compare the capture of maternal condition in the existing ICD-PM with the maternal codes from the WHO application of ICD-10 to death during pregnancy, childbirth, and the puerperium to explore benefits in the quality of data received	Data was already used in another eligible article
**Allanson, 2016**	Application of ICD-PM to pretermrelated neonatal deaths in South Africa and United Kingdom	Population based, retrospective application to two perinatal death databases (UK and South Africa)	To explore preterm-related neonatal deaths using the WHO application of the International Classification of Disease (ICD-10) to death during the perinatal period.	Data was already used in another eligible article
**Aminu, 2017**	Cause of and factors associated with stillbirth: a systematic review of classification systems.	Systematic review	A systematic review of stillbirth classification systems to highlight their strengths and weaknesses for practitioners and policymakers.	Comparison of classification systems, but no original ICD-PM data
**Aminu, 2019**	Stillbirth in low- and middleincome countries: addressing the 'silent epidemic'	Literature review	We highlight where and how focused interventions and implementation research is needed and would be effective	No reporting of primary data
**Anguinaga, 2021**	Causal analysis of fetal death in high-risk pregnancies	Retrospective cohort	To determine the causes of fetal death among the stillbirths using two classification systems from 22 weeks of gestation in a period of three years in high-risk pregnancies.	The ICD-PM data could not be extracted from the manuscript or supplementary files, and the article did not include information on the use of the tool

**Auger, 2020**	Stillbirth Among Arab Women in Canada, 1981-2015	Retrospective study	We examined inequality in stillbirth rates between Arab women and the French and English majority of women in Quebec, Canada	Use of ICD-10 Codes, but not the ICD-PM
**Bobona, 2017**	A case-control study of causes of and factors associated with stillbirth at the Port Moresby General Hospital, Papua New Guinea	Case control study	To re-evaluate the causes of and risk factors associated with stillbirths so as to recommend strategies to further reduce the 'static' stillbirth rates in our institution	No reporting of primary data (Conference Abstract)
**Baptista, 2021**	Evolution of neonatal mortality by preventable causes over the past ten years in Espírito Santo State	Ecological study	Analyze neonatal mortality causes, highlighting the preventable ones from 2008 to 2017, in Espírito Santo State and health regions.	Use of ICD-10 Codes, but not the ICD-PM
**Best, 2019**	Assessing the deprivation gap in stillbirths and neonatal deaths by cause of death: a national population-based study	Retrospective Cohort	To investigate socioeconomic inequalities in cause-specific stillbirth and neonatal mortality to identify key areas of focus for future intervention strategies to achieve government ambitions to reduce mortality rate	Classification other than ICD-PM
**Bogale, 2017**	Why gone too soon? Examining social determinants of neonatal deaths in northwest Ethiopia using the three-delay model approach	Community based social autopsy of 39 neonatal deaths	To investigate the delays in care seeking that are associated with newborn deaths in northwest Ethiopia using the three-delay model approach	Classification other than ICD-PM
**Çama, 2021**	Infant mortality rates and causes of infant deaths in Kilis province between 2012 and 2018	Retrospective, cross-sectional study	To determine the infant mortality rate and the causes of infant death in Kilis Province, Turkey	Use of ICD-10 Codes, but not the ICD-PM
**Carlo, 2016**	Maternal and neonatal mortality: time to act	Editorial	Literature review of maternal and neonatal mortality	No reporting of primary data
**Danilova, 2016**	Identifying potential differences in cause-of-death coding practices across Russian regions	Cross sectional study based on population-based mortality data	To evaluate the uniformity of cause-ofdeath coding practices across Russian regions using an indirect method.	Classification other than ICD-PM (and causes of all deaths, not perinatal deaths specifically)
**Duby, 2020**	Effect of an integrated neonatal care kit on cause-specific neonatal mortality in rural Pakistan.	a community-based, cluster-randomized controlled trial of 5286 neonates in Rahim Yar Khan (RYK), Punjab, Pakistan between April 2014 and August 2015.	To describe the causes of neonatal death in a rural area in Pakistan and to estimate the effect of an integrated neonatal care kit (iNCK) on causespecific neonatal mortality	Use of ICD-10 Codes, but not the ICD-PM
**Flenady, 2016**	Stillbirths: recall to action in highincome countries.	Literature review	This Series paper reviews the status of stillbirths worldwide and the progress since the Lancet Stillbirths Series in 2011	No data on the causes of perinatal death

**Flenady, 2020**	Classification of causes and associated conditions for stillbirths and neonatal deaths.	Literature review	To review the classification of perinatal death, the contemporary classification systems including the World Health Organization's International Classification of Diseases - Perinatal Mortality (ICD-PM), and next steps.	Comparison of classification systems, but no original ICD-PM data
**Goldenberg, 2018**	Cause of stillbirth reporting	Commentary	Reviewing cause of stillbirth reporting	No reporting of primary data
**Grandi, 2018**	Neonatal mortality in the framework of the Millennium Development Goals and new post2015 goals	Commentary	Reviewing neonatal mortality	No reporting of primary data
**Hatzistergos, 2021**	Review of stillbirths at a tertiary hospital over a six-year period	Retrospective cross-sectional stu y	To compare our rate and cause of stillbirths to the national data and to investigate our compliance to undertaking the investigations re uired to diagnose the cause of stillbirth	Classification other than ICD-PM
**Hauksdottir, 2018**	Perinatal mortality in Iceland 1988 - 2017	Retrospective cross-sectional stu y	To evaluate how perinatal mortal ty rate and its causes have changed n i Iceland during the last 30 years, particularly to see if it is possible to lower the perinatal mortality rate even more.	Classification other than ICD-PM
**Higgings, 2018**	Persistent inaccuracies in completion of medical certificates of stillbirth: a cross-sectional study	Cross-sectional study	A repeat cross-sectional audit to ssess whether practice had improved (previous demonstrated widespre d inaccuracies in MCS completion) following introduction of a regio al care pathway	Classification other than ICD-PM
**Huicho, 2016**	Examining national and districtlevel trends in neonatal health in Peru through an equity lens: a success story driven by political will and societal advocacy	Cross-sectional study	To: (a) describe national and district NMR variations over time; (b) as ess NMR trends by wealth quintile a d of place of residence: (c) describe ty; evolution mortality causes; (d) ed assess completeness of registered mortal (e) assess coverage and equity of NMR-rela interventions; and (f) explore underlying driving factors.	Classification other than ICD-PM
**Jahani, 2016**	Prevalence and Etiology of Perinatal Period Mortality in Hospitals, Iran	Cross-sectional study	To determine the prevalence and g the etiologic factors of mortality duri n. perinatal period at hospitals in Ira	Use of ICD-10 Codes, but not the ICD-PM
**Kapurubandara, 2017**	A perinatal review of singleton stillbirths in an Australian metropolitan tertiary center	A retrospective case series	To investigate an apparent rise in stillbirths at a Sydney tertiary ref rral hospital in Australia	Classification other than ICD-PM

**Kirby, 2016**	Optimizing the ICD to identify the maternal condition in the cause of perinatal death: overcoming challenges to create a holistic approach	Commentary	Reviewing maternal conditions associated with perinatal death	No reporting of primary data
**Kortekaas, 2018**	Perinatal Death beyond 41 weeks pregnancy: an evaluation of causes and substandard care factors as identified in perinatal audit in the Netherlands	Cross sectional study using Perinatal Audit Registry of the Netherlands (PARS)	We evaluated causes of death and substandard care factors (SSFs) in term and post term perinatal death	Classification other than ICD-PM
**Lai, 2019**	Stillbirth and perinatal death: a retrospective study with application of ICD-PM to autopsies performed between 2007 and 2018 in Coimbra	Retrospective study	Identify main changeable causes of perinatal death	No full text available
**Lanas, 2021**	Understanding life and death in Latin America	Literature review	Evaluation of death causes in Latin America	No reporting of primary data
**Lawn, 2016**	Stillbirths: rates, risk factors, and acceleration towards 2030.	Literature review	This Series paper reviews the status of stillbirths worldwide and the progress since the Lancet Stillbirths Series in 2011	No data on the causes of perinatal death
**Liu, 2021**	Neonatal mortality and leading causes of deaths: a descriptive study in China, 2014 – 2018	Cross sectional study based on data from the National Maternal and Child Health Surveillance System.	The present study estimated the national and urban-rural levels and causes of neonatal deaths in china annually between 2014 and 2018 to provide data support for the further end of preventable neonatal deaths for China and other low-income and middle-income countries	Use of ICD-10 Codes, but not the ICD-PM
**Lu, 2016**	Neonatal mortality in the urban and rural China between 1996 – 2013: a retrospective study	Retrospective study	To examine changes in neonatal mortality rate (NMR) and causespecific NMR in urban and rural areas to guide renewed efforts to further reduce NMR in China	Use of ICD-10 Codes, but not the ICD-PM
**Madhi, 2019**	Causes of stillbirths among women from South Africa: a prospective, observational study.	Prospective, observational study	To investigate the causes of stillbirths in fetuses of at least 22 weeks' gestational age or with a birthweight of at least 500 g.	Classification other than ICD-PM
**Maducolil, 2018**	Risk factors and classification of stillbirth in a Middle Eastern population: a retrospective study	Retrospective cohort	To estimate the incidence of stillbirth, explore the associated maternal and fetal factors and to evaluate the most appropriate classification of stillbirth for a multiethnic population.	The ICD-PM data could not be extracted from the manuscript or supplementary files, and the article did not include information on the use of the tool
**Man, 2016**	Stillbirth and intrauterine fetal death: factors affecting determination of cause of death at autopsy.	Retrospective cross-sectional study of detailed autopsy reports from intrauterine deaths in the second and third trimesters during 2005 – 2013, to assign a cause of death using predefined objective criteria.	This study aimed to examine factors relating to determination of CoD using a large dataset from two specialist centers in which observer bias had been reduced by classifying findings objectively and assigning CoD based on predetermined criteria.	Classification other than ICD-PM

**Man, 2016**	Stillbirth and intrauterine fetal death: role of routine histopathological placental findings to determine cause of death.	Retrospective-cross sectional study: a dedicated database was used to collate antenatal and postmortem examination details for all cases examined between 2005 and 2013 at two tertiary specialist centers in London, UK.	To examine objectively classified placental findings from a series of > 1000 autopsies following intrauterine death in order to evaluate the role of placental histological examination in determining the cause of death.	Classification other than ICD-PM
**McClure, 2018**	Global Network for Women's and Children's Health Research: probable causes of stillbirth in low- and middle-income countries using a prospectively defined classification system	Prospective, observational study	We sought to classify causes of stillbirth for six low-middle-income countries using a prospectively defined algorithm.	Classification other than ICD-PM
**Miranda, 2020**	The Importance of Autopsy in Early Neonatal Death in Portugal	Retrospective study	To characterize early neonatal death and the clinical importance of autopsy	Classification other than ICD-PM
**Monmany, 2021**	Extended Perinatal Mortality Audit in a Rural Hospital in India	Retrospective Cohort	The aim of the study is to describe the status of perinatal mortality (PM) in an Indian rural hospital.	The ICD-PM data could not be extracted from the manuscript or supplementary files, and the article did not include information on the use of the tool
**Moran, 2018**	Implementation of maternal and perinatal death surveillance and response	Conference abstract / Editorial	1. To present MPDSR TWG guidance and tools and 2. To Share experiences successes and barriers of MPDSR implementation in three countries	No reporting of primary data
**Motovic, 2016**	Perinatal mortality at the maternity hospital in Zajecar in 2015	Cross sectional	To determine perinatal mortality at the Maternity Hospital in Zajecar in 2015	Classification other than ICD-PM
**Norris, 2017**	Causes and temporal changes in nationally collected stillbirth audit data in high resource	Literature review	In this article, stillbirth rates over recent years are reviewed together with changes in the classification of the cause of death of stillbirths.	Classification other than ICD-PM
**O'Farrell, 2017**	Stillbirth in Ireland, 2015	Conference abstract	Not clearly stated in abstract	No reporting of primary data
**Ovalle, 2016**	A new fetal death classification system	Retrospective Cohort	To report a system for classification of fetal deaths	Classification other than ICD-PM
**Priyani, 2017**	Classification of perinatal deaths according to ICD-PM: An audit on perinatal post-mortems in a tertiary care centre in Sri Lanka	Retrospective Cohort	This paper gives data of a post-mortem based study, classifying causes of stillbirths, intra-partum deaths and neonatal deaths according to ICD-PM, and identifying the commonly occurring congenital malformations related to organ systems in stillbirths, intra partum deaths and neonatal deaths, and their associated risk factors.	The ICD-PM data could not be extracted from the manuscript or supplementary files, and the article did not include information on the use of the tool
**Reinebrant, 2017**	Global reporting of the causes of stillbirth: A systematic review	Conference abstract	To comprehensively summarize the causes of stillbirth reported globally to identify areas for prevention and improvement in data quality	No reporting of primary data
**Reinebrant, 2018**	Making stillbirths visible: a systematic review of globally reported causes of stillbirth	Systematic review	To identify globally reported causes of stillbirth, classification systems and alignment with the ICD-PM	No reporting of primary data
**Reinebrant, 2018**	Validity of verbal autopsy for ascertaining the causes of stillbirth	Prospective cohort	To validate the verbal autopsy tool for stillbirths of the World Health Organization (WHO) by using hospital diagnosis of the underlying cause of stillbirth (golden standard) and to compare the fraction of stillbirths attributed to various specific causes through hospital assessment versus verbal autopsy	Classification other than ICD-PM

**Rêgo, 2018**	Perinatal deaths preventable by intervention of the Unified Health System of Brazil	Descriptive study	To describe the epidemiological characteristics of perinatal deaths through the actions of the Unified Health System	Classification other than ICD-PM
**Roos, 2016**	Learning from every stillbirth and neonatal death	Commentary	Not clearly stated	No reporting of primary data
**Roro, 2019**	Predictors, causes and trends of neonatal mortality at Nekemte Referral Hospital, east Wollega Zone, western Ethiopia (2010-2014)	Retrospective Cohort	To assess predictors, causes and trends of neonatal mortality amongst neonates admitted to Nekemte Referral Hospital neonatal unit between 2010 and 2014	Classification other than ICD-PM
**Silver, 2020**	The enemy of the good in assigning cause of fetal death	Commentary	Not clearly stated	No reporting of primary data
**Smith, 2020**	Review of Stillbirth in a Canadian Tertiary Care Centre.	Retrospective chart review	To determine the causes of stillbirth in a Canadian tertiary care centre and to identify the risk factors present in these deliveries	Classification other than ICD-PM
**Turowski, 2017**	Classification system for placental abnormalities and the importance of stillbirth classification	No abstract or full text available	No abstract or full text available	No full text available
**Valentine, 2020**	Neonatal mortality rates in neonatal intensive care unit at Kamuzu central hospital in Malawi	Prospective, observational study	To evaluate the neonatal mortality rate and causes of death in a large, tertiary, referral hospital, Kamuzu Central Hospital in Lilongwe, Malawi	Classification other than ICD-PM
**Vallely, 2021**	Perinatal death audit and classification of stillbirths in two provinces in Papua New Guinea: A retrospective analysis	Retrospective cross-sectional	To undertake a retrospective perinatal death audit and assessment of avoidable factors associated with stillbirths among a cohort of women in two provinces in Papua New Guinea	Classification other than ICD-PM
**Wasim, 2017**	Perinatal death audit using WHO 2016 audit guide: ICD-PM making every baby count	Conference abstract	Not clearly stated	No reporting of primary data
**Worrel, 2019**	Stillbirth burden and challenges for reporting: Initial results from the champs makeni site, Sierra Leone	Conference abstract?	Not clearly stated	No reporting of primary data
**Yan, 2021**	Neonatal deaths among infants born to woman living with HIV in the UK and Ireland 1998 - 2017	Population-based surveillance	To estimate the incidence of neonatal mortality among infants born to women living with HIV in the UK and Ireland in 1998-2017, describe causes of neonatal death (NND) and examine risk factors.	The ICD-PM data could not be extracted from the manuscript or supplementary files, and the article did not include information on the use of the tool
**Zulfeen, 2021**	*What do the numbers say?* – Introduction of the WHO ICD-PM classification and fetuses-at risk approach in perinatal audit, South India	Prospective cohort	The aim of this study was to perform a qualitative perinatal audit and devise methods for future audits	The ICD-PM data could not be extracted from the manuscript or supplementary files, and the article did not include information on the use of the tool

Supplementary file 3. Data collection form

Notes on using a data extraction form:

- Be consistent in the order and style you use to describe the information for each included study.
- Record any missing information as unclear or not described, to make it clear that the information was not found in the study report(s), not that you forgot to extract it.
- Include any instructions and decision rules on the data collection form, or in an accompanying document. It is important to practice using the form and give training to any other authors using the form.
- You will need to protect the document in order to use the form fields (Tools / Protect document)

**Review title or ID**

**Study ID** *(surname of first author and year first full report of study was published e.g. Smith 2001)*

**Report IDs of other reports of this study** *(e.g. duplicate publications, follow-up studies)*

**Notes:**

1. General Information

Date form completed *(dd/mm/yyyy)*	
Name/ID of person extracting data	
Report title *(title of paper/ abstract/ report that data are extracted from)*	
Report ID *(if there are multiple reports of this study)*	
Reference details	
Report author contact details	
Publication type *(e.g. full report, abstract, letter)*	
Study funding source *(including role of funders)*	
**Possible conflicts of interest** *(for study authors)*	
Notes:	

2. Eligibility

**Study Characteristics**	**Review Inclusion Criteria** *(Insert inclusion criteria for each characteristic as defined in the Protocol)*	**Yes/ No / Unclear**	**Location in text** *(pg & ¶/fig/table)*

Type of study	Prospective application of ICD-PM	…	
**Study Characteristics**	**Review Inclusion Criteria** *(Insert inclusion criteria for each characteristic as defined in the Protocol)*	**Yes/ No / Unclear**	**Location in text** *(pg & ¶/fig/table)*
	Retrospective application of ICD-PM	…	
	Comparison of classification studies	…	
	Other design (specify):	…	
Type of deaths included	Perinatal deaths (from .. weeks of pregnancy/… grams of birthweight till … week(s) after birth)	…	
	Neonatal deaths (till… week(s) after birth)		
	Stillbirths (from … weeks of pregnancy/… grams birthweight)		
Types of classification	ICD-PM only	…	
	ICD-PM and … (other classification(s) used)	…	
	Classification other than ICD-PM (specify):		
Types of outcome measures	**Primary outcome measure**<br><br>**Secondary outcome measure**	…	
Decision:	…		
Reason for exclusion			
Notes:			

3. Population and setting

	**Description** *Include comparative information for each group (i.e. intervention and controls) if available*	**Location in text** *(pg & ¶/fig/table)*
Population description *(from which study participants are drawn)*		
Setting *(including location and social context)*		
Inclusion criteria		
Exclusion criteria		

Method/s of recruitment of participants		
Notes:		

4. Methods

	**Descriptions as stated in report/paper**	**Location in text** *(pg & ¶/fig/table)*
Aim of study		
Design *(e.g. parallel, crossover, non-RCT)*		
Selection of cases *(hospital data, vital registration etc.)*		
Method of classification (e.g. confidential enquiry, one author, two authors)		
Information availability (paper/electronic medical files, postmortem laboratory/radiology, autopsy)		
Start date		
End date		
Notes:		

5. Risk of Bias assessment

See the Appraisal tool for Cross-Sectional Studies (AXIS, 2016)

6. Participants

Provide overall data and, if available, comparative data for each intervention or comparison group.

	**Description as stated in report/paper**	**Location in text** (pg & ¶/fig/table)
Total no. of perinatal deaths for which ICD-PM was applied<br>Number of stillbirths<br>Number of neonatal deaths		
Total no. of perinatal deaths for which other classification was applied<br>Number of stillbirths<br>Number of neonatal deaths		
Baseline imbalances		
Withdrawals and exclusions (if not provided below by outcome)		

Setting<br>(high- middle- low-income)		
Setting<br>(rural, urban, suburban or mixed)		
Maternal age		
Parity		
Maternal Race/Ethnicity		
	**Description as stated in report/paper**	**Location in text**<br>(pg & ¶/fig/table)
Place of ANC		
Number of ANC visits		
Other relevant sociodemographic characteristics		
Sex baby		
Subgroups measured		
Subgroups reported		
Notes:		

7. Results

	**Description as stated in report/paper**						**Location in text** *(pg & ¶/fig/table)*
Timing of death<br>:	**Timing (n) %)**						
	Antepartum		Intrapartum		Postpartum		
**Cause of Death**	**Maternal condition**						
Antepartum Deaths<br>A1<br>A2<br>A3<br>A4<br>A5<br>A6	M1 N (%)	M2	M3	M4	M5	M6	
Intrapartum Deaths<br>I1<br>I2<br>I3<br>I4<br>I5<br>I6<br>I7	M1	M2	M3	M4	M5	M6	

Neonatal Deaths<br>N1<br>N2<br>N3<br>N4<br>N5<br>N6<br>N7<br>N8<br>N9<br>N10<br>N11	M1	M2	M3	M4	M5	M6	

Copy and paste the appropriate table for each outcome, including additional tables for each time point and subgroup as required.

	**Description as stated in report/ paper**		**Location in text** *(pg & ¶/fig/table)*
Ratios<br>Perinatal death ratio<br>Stillbirth ratio<br>Neonatal death ratio			
No. missing participants and reasons	n…. (% of total incl)		Reason…
Challenges/Improvements for ICD-PM reported	Yes/no		
If yes, note challenges	1. ….<br>2. ….		
If yes, note improvements	1. ….<br>2. …..		
Any other results reported			
Notes:			

8. Applicability

Have important populations been excluded from the study?<br>*(consider disadvantaged populations, and possible differences in the intervention effect)*	...<br>*Yes/No/Unclear*	
Is the intervention likely to be aimed at disadvantaged groups?<br>*(e.g. lower socioeconomic groups)*	...<br>*Yes/No/Unclear*	
Does the study directly address the review question?<br>*(any issues of partial or indirect applicability)*	...<br>*Yes/No/Unclear*	
Notes:		

9. Other information

	**Description as stated in report/paper**	**Location in text** *(pg & ¶/fig/table)*
Key conclusions of study authors		
References to other relevant studies		
Correspondence required for further study information *(what and from whom)*		
Further study information requested *(from whom, what and when)*		
Correspondence received *(from whom, what and when)*		
Notes:		

Supplementary Table 4. Quality assessment of included articles according to the AXIS tool

	**Introduction**	**Methods**										**Results**					**Discussion**		**Other**		**Total points**	**score**	**Quality**
Author, year	Aims	Study design	Sample size	Study population	Representativity	Selection process	Measurements for nonresponders	Appropriate risk factors and outcomes	Measurement of risk factors and outcomes	Statistical significance	repeatability	Data description	Response rate / missing deaths	Nonresponders	consistency	Completeness of analysis	Justification	Limitations	Conflicts of interest	Ethical approval			
**Miyoshi, 2019**	1	1	N/A	0	0	0	N/A	1	1	0	1	1	0	N.A.	1	0	0	1	1	1	10/17	0·59	Moderate
**Wasim, 2020**	1	1	N/A	1	1	1	N/A	1	1	1	1	1	0	N.A.	1	1	0	1	1	0	14/17	0·82	Strong
**Lavin, 2018**	1	1	N/A	1	1	1	N/A	1	0	0	1	1	0	N.A.	1	0	1	0	1	1	12/17	0·71	Moderate-Strong
**Madhi, 2019[1]**	1	0	N/A	0	0	0	N/A	1	1	1	1	1	0	0	1	0	1	1	0	1	10/18	0·56	Moderate
**Dase, 2020**	1	1	N/A	0	1	1	N/A	1	1	1	0	1	0	N.A.	1	1	1	1	1	1	14/17	0·82	Strong
**Prüst, 2020**	1	1	N/A	1	1	1	N/A	1	1	1	1	1	0	N.A.	1	1	1	1	1	0	15/17	0·88	Strong
**Aminu, 2019**	1	1	1	1	1	0	1	1	1	1	1	1	0	N.A.	1	1	1	0	1	0	15/19	0·79	Moderate-Strong
**Allanson, 2016**	1	0	N/A	0	1	0	N/A	1	0	0	0	1	0	N.A.	1	0	1	0	0	1	7/17	0·41	Weak
**Housseine, 2021**	1	1	N/A	0	1	1	N/A	1	1	0	1	1	0	N.A.	0	1	1	1	1	1	13/17	0·76	Moderate-Strong
**Salazar-Barrientos, 2019**	1	1	N/A	1	1	1	N/A	1	1	0	1	1	0	N.A.	1	1	1	1	0	1	14/17	0·82	Strong
**Fabrizio, 2020**	1	1	N/A	1	1	1	N/A	1	1	1	1	1	0	N.A.	1	1	1	1	1	0	15/17	0·88	Strong
**Sharma, 2021**	1	1	N/A	1	1	1	N/A	1	1	0	1	1	0	N.A.	1	1	1	1	1	1	15/17	0·88	Strong
**Luk, 2020**	1	1	N/A	1	0	1	N/A	1	1	1	0	1	0	N.A.	1	1	1	1	1	1	14/17	0·82	Strong
**Shattnawi, 2020**	1	0	N/A	1	0	1	N/A	1	1	1	1	1	1	N.A.	1	1	1	1	1	0	14/17	0·82	Strong

Legend
[1]Inclusion of both articles
[1,2]

Supplementary Table 5. Elaborated table on the causes of perinatal deaths according to the ICD-PM, per income setting

		High-income settings			Middle-income settings			Low- income settings		
Number of studies		3			9			4		
Countries		United Kingdom, Italy, Hong Kong			South-Africa, Colombia, Suriname, Jordan, Pakistan, India			Sierra-Leone, Zimbabwe, Kenya, Malawi, Nigeria, Zambia, Tanzania		
***Inclusions, n=**		**9629**			**32715**			**2556**		
		**N =**	**Median %**	**Range %**	**N =**	**Median %**	**Range %**	**N =**	**Median**	**Range**
**Main causes Antepartum deaths N =**	**Total**	**4880**			**17897**			**825**		
	**A1 Congenital malformations, deformations and chromosomal abnormalities**	1014	**21%**	6 - 22	715	**4%**	2 – 20	23	**3 %**	2 – 14 %
	**A2 Infection**	42	**1%**	0·05 - 8	529	**3%**	0·3 – 44	30	**4 %**	0 – 9 %
	**A3 Antepartum hypoxia**	249	**5%**	0 - 55	1159	**6%**	0 – 62	95	**12 %**	0 – 46 %
	**A4 Other specified antepartum disorder**	122	**3%**	2 - 4	3048	**17%**	0·8 – 19	2	**0 %**	0 – 0·3 %
	**A5 Disorder related to foetal growth**	688	**14%**	6 – 19	1595	**9%**	0·8 – 20	207	**25 %**	0 – 57 %
	**A6 Foetal death of unspecified cause**	2765	**57%**	22 – 60	10851	**61%**	2 – 68	468	**57 %**	30 – 89 %
**Main causes Intrapartum deaths N =**	**Total**	**488**			**4390**			**1192**		
	**I1 Congenital malformations, deformations and chromosomal abnormalities**	15	**3%**	3 – 100	300	**7%**	3 – 29	50	**4 %**	2 – 16 %
	**I2 Birth trauma**	5	**1%**	0 - 1	1	**0%**	0 – 0.3	0	**0 %**	0 %
	**I3 Acute intrapartum event**	314	**64%**	0 - 65	2951	**67%**	0 – 94	350	**29 %**	10 – 84 %
	**I4 Infection**	5	**1%**	0 – 17	62	**1%**	0 – 22	23	**2 %**	0 – 4 %
	**I5 Other specified intrapartum disorder**	1	**0%**	0 – 0·2	489	**11%**	0 – 29	2	**0 %**	0 – 0.3 %
	**I6 Disorders related to foetal growth**	25	**5%**	0 – 5	181	**4%**	0 – 49	157	**13 %**	0 – 40 %
	**I7 Intrapartum death of unspecified cause**	123	**25%**	0 – 26	406	**9%**	0 – 43	610	**51 %**	0 – 61 %
**Main causes Neonatal deaths N =**	**Total**	**4260**			**10058**			**291**		
	**N1 Congenital malformations, deformations and chromosomal abnormalities**	1163	**27%**	15 – 27	1276	**13%**	5 – 29	20	**7 %**	2 – 8 %
	**N2 Disorder related to foetal growth**	15	**0%**	0·3 – 4	161	**2%**	0 – 4	1	**0 %**	0 – 0·4 %
	**N3 Birth trauma**	11	**0%**	0 – 0·3	3	**0%**	0 – 0·2	0	**0 %**	0 %

	**N4 Complications of intrapartum events**	75	**2%**	2 – 4	2325	**23%**	0 – 29	117	**40 %**	40 – 44 %
	**N5 Convulsions and disorders related to cerebral status**	26	**1%**	0 – 1	134	**1%**	0 – 26	0	**0 %**	0 %
	**N6 Infection**	66	**2%**	2 – 15	722	**7%**	2 – 27	16	**5 %**	5 – 7 %
	**N7 Respiratory and cardiovascular disorders**	288	**7%**	7 – 11	1991	**20%**	3 – 35	37	**13 %**	5 – 14 %
	**N8 Other neonatal conditions**	108	**3%**	2 – 19	611	**6%**	1 – 10	3	**1 %**	0 – 1 %
	**N9 Low birthweight and prematurity**	1347	**32%**	33 – 32	2611	**26%**	10 – 53	38	**13 %**	9 – 37 %
	**N10 Miscellaneous**	20	**0%**	0 – 0·5	178	**2%**	0 – 2	1	**0 %**	0 – 0·4 %
	**N11 Neonatal death of unspecified cause**	1141	**27%**	0 – 27	46	**0%**	0 – 2	58	**20 %**	5 – 23 %
**Unknown timing, n=**		**1**			**370**			**248**		
**Main Maternal condition N =**	**Total**	**9629**			**32195**			**2556**		
	**M1 Complications of placenta, cord and membranes**	2543	**26%**	24 – 71	5565	**17%**	13 – 34	585	**23 %**	6 – 27
	**M2 Maternal complications of pregnancy**	987	**10%**	2 – 15	1268	**4%**	2 – 13	204	**8 %**	6 – 10
	**M3 Other complications of labour and delivery**	760	**8%**	0·5 – 8	4485	**14%**	2 – 18	473	**19%**	9 – 44
	**M4 Maternal medical and surgical conditions**	734	**8%**	7 – 22	9637	**30%**	6 – 50	587	**23%**	4 – 42
	**M5 No maternal condition identified**	4605	**48%**	16 – 50	11148	**35%**	16 – 57	707	**28%**	9 – 55
	Maternal condition not classified				*612*					

References

1. Madhi SA, Pathirana J, Baillie V, et al. An Observational Pilot Study Evaluating the Utility of Minimally Invasive Tissue Sampling to Determine the Cause of Stillbirths in South African Women. *Clin Infect Dis* 2019; **69** (suppl 4): S342–50.
2. Madhi SA, Pathirana J, Baillie V, et al. Unraveling Specific Causes of Neonatal Mortality Using Minimally Invasive Tissue Sampling: An Observational Study. *Clin Infectious Diseases* 2019; **69** (suppl 4): S351–6.
